# Successful Treatment of Invasive Mucormycosis in Orthotopic Liver Transplant Population

**DOI:** 10.1155/2021/8667589

**Published:** 2021-12-06

**Authors:** Taryn A. Eubank, Constance M. Mobley, Mozhgon Moaddab, Mark J. Hobeika, Melissa O'Neal, William L. Musick, Joshua M. Knight, Joseph S. Galati, Sudha Kodali, Akshay Shetty, David W. Victor, Ashish Saharia, R. Mark Ghobrial, Kevin A. Grimes

**Affiliations:** ^1^Department of Pharmacy, Houston Methodist Hospital, Houston, TX, USA; ^2^Department of Surgery, Houston Methodist Hospital, Houston, TX, USA; ^3^JC Walter Jr Transplant Center, Houston Methodist Hospital, Houston, TX, USA; ^4^Sherrie and Alan Conover Center for Liver Disease & Transplantation, Houston, TX, USA; ^5^Weill Cornell College of Medicine, New York, NY, USA; ^6^Department of Medicine, Houston Methodist Hospital, Houston, TX, USA; ^7^Department of Hepatology, Houston Methodist Hospital, Houston, TX, USA; ^8^Division of Infectious Diseases, Houston Methodist Hospital, Houston, TX, USA; ^9^Houston Methodist Research Institute, Houston, TX, USA

## Abstract

Mucormycosis is caused by ubiquitous fungi and encompasses a variety of different opportunistic syndromes in humans that disproportionately affect immunocompromised patients. Mortality has been documented to range between 50 and 100%; however, location of infection greatly dictates likelihood of survival. Treatment of mucormycosis involves aggressive surgical intervention and combination therapy of antifungal agents. In solid organ transplant recipients, immunosuppressive agents used to prevent rejection of the transplanted organ pose additional obstacles in the treatment of invasive fungal infections. We report on 3 high models for end-stage liver disease (MELD-Na) score orthotopic liver transplant (OLT) recipients who all were diagnosed with *Rhizopus* spp. infections with positive, 1-year outcomes after aggressive, individualized treatment.

## 1. Introduction

Mucormycosis represents a group of filamentous fungi that belong to the order Mucorales which causes life-threatening infections. Immunocompromised hosts, such as patients with immunosuppressive therapy associated with transplantation, poorly controlled diabetes mellitus, prolonged neutropenia, and high-dose corticosteroid treatment, are at increased risk [1]. Fungi within the genus *Rhizopus* are reported as the most predominant cause of mucormycosis human infections [2]. Diagnosis and treatment are challenging due to rapid progression, suboptimal culture recovery, and diminished tissue perfusion thus decreased antifungal penetration. In addition, many antifungal agents have troublesome side effects and/or drug-drug interactions that can be an obstacle during treatment. It is due to these issues that aggressive surgical intervention in combination with targeted antifungals is imperative for treatment success.

## 2. Case Reports

### 2.1. Patient 1

**Figure 1:**
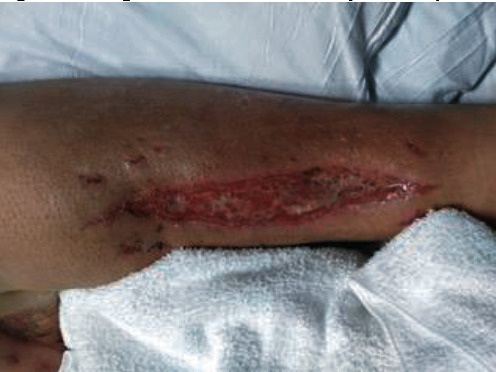
Image of cutaneous mucormycosis in patient 1 status postfasciotomy.

The first patient is a 34-year-old Caucasian female with a past medical history consisting of autoimmune hepatitis, type 1 diabetes mellitus requiring insulin pump, end-stage renal disease, history of deep vein thrombosis, peripheral vascular disease with femoral popliteal bypass, and extensive history of solid organ transplantations (previously underwent 6 organ transplants consisting of 3 kidneys and 3 livers) with the most recent being a simultaneous liver-kidney transplant 20 months prior to presentation. The patient was being maintained on tacrolimus, mycophenolate mofetil, and prednisone for immunosuppression (Table 1). Due to loss of previous graft from rejection, she was maintained on a high-dose triple drug immunosuppressive regimen to prevent recurrence of rejection. One month prior to diagnosis, she presented with acute left leg pain and swelling felt to be secondary to chronic pain from hardware repair of extremity several years ago. She continued to experience extremity pain and was subsequently admitted. On this admission, the patient was taken to the operating room (OR) for fasciotomy (Figure 1). No necrotic tissue was found, and she was sent home with a wound vacuum. Upon follow-up with her outpatient wound clinic, fat necrosis was noted on the affected leg and a debridement was completed. A rapid genetic sequencing swab (MicroGen Diagnostics©) was run at the clinic showing 94% *Enterococcus faecalis*, 93% *Rhizopus oryzae*, and 5% *Aspergillus flavus*. The patient presented to our hospital for further work up of polymicrobial infection and potential invasive fungal disease.

On hospital day 2, she was started on intravenous (IV) isavuconazole 372 mg daily and returned to the OR for debridement. Preliminary tissue, fungal, and anaerobic culture reports from the OR grew diphtheroids, *Klebsiella oxytoca*, *Enterococcus faecalis*, and an unidentified mould. On hospital day 5, the cultures identified the mould as *Rhizopus* spp. Intravenous liposomal amphotericin B 3 mg/kg daily was added. The same day she went back to the OR for debridement, and a biopsy sample was sent to pathology. The pathology report confirmed organisms consistent with mucormycosis. On hospital day 15, she went back to the OR for another serial debridement and biopsy. That same day, fungal susceptibilities returned, displaying isavuconazole resistance (Table 2). She was switched to oral posaconazole 300 mg daily and IV micafungin 150 mg daily in addition to amphotericin B. Three days later, her wound vacuum was replaced, and the pathology report showed multifocal fungal elements seen on a Grocott-Gomori's methenamine silver stain consistent with clinical history of ‘Mucor/Rhizopus.' There was necrotic tissue with no definite involvement of vessels or margins. Dermal and subcutaneous tissue displayed necrosis, hemorrhage, fibrosis, granulation tissue, and foreign body giant cell reaction. The wound vacuum was left in place, and on hospital day 20, another wound culture grew mould. At this point, given the aggressive nature of the disease, it was decided to utilize amphotericin B deoxycholate irrigation in the wound vacuum and continue systemic antimicrobials. Amphotericin B 50 mg in 1000 mL sterile water for irrigation was added to the Veraflo® wound vacuum, and pulse lavages were administered to the affected area twice daily. Nine days later, the patient went back to the OR for another debridement and repeat pathology report from that sample showed no evidence of fungus. The patient received amphotericin wound vacuum irrigation with pulse lavages for a total of 20 days. She was discharged on oral posaconazole 300 mg every 8 hours and ascorbic acid 1000 mg tablet daily to help with absorption and therapeutic drug monitoring (Table 3) with plans to follow-up for the skin graft of the area. The patient remained *Rhizopus* spp. free at 1-year postdiagnosis.

### 2.2. Patient 2

Patient 2 is a 58-year-old Caucasian female with past medical history significant for obesity and upper gastrointestinal bleed who underwent an OLT secondary to chronic hepatitis C virus (HCV) infection and alcoholic cirrhosis. MELD-Na at time of transplant was 42. There were no immediate complications that occurred throughout the procedure. Immunosuppression consisting of a corticosteroid taper, tacrolimus, and mycophenolate mofetil (Table 1) was started on day of transplant per institution protocol. Infection prophylaxis consisted of sulfamethoxazole/trimethoprim (POD3), valganciclovir (POD1), and voriconazole (started 5 days prior to transplantation per protocol for high MELD-Na pretransplant patients in intensive care units) [3].

**Figure 2:**
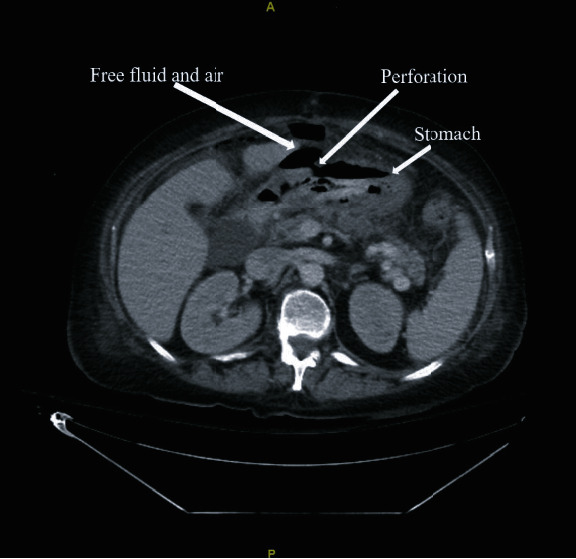
Patient 2 CT abdomen exhibiting bowel perforation secondary to mucormycosis.

On POD10, department of infectious diseases was consulted for persistent leukocytosis and abdominal cramping. Notable findings included an endoscopy exhibiting ulcerations and old blood on POD18 and an indium scan exhibiting increased uptake in the abdomen; however, leukocytosis resolved and the patient was discharged to inpatient rehabilitation. Patient was then readmitted on POD30 for increased abdominal pain, and a computed tomography (CT) scan exhibited free air in her abdomen suspicious for gastric perforation (Figure 2). The patient was emergently taken to the OR for exploratory laparotomy with findings of a large necrotic ulcer in the antrum causing spillage of gastric contents. An OR biopsy sample resulted in *Rhizopus* spp., and on POD33, the patient was initiated on IV liposomal amphotericin B 3 mg/kg daily, micafungin 150 mg daily, and isavuconazole 372 mg every 8 hours. After 48 hours of therapy, the patient was transitioned from isavuconazole 372 mg every 8 hours to daily. In conjunction with the antifungal regimen, weekly OR debridement and washouts were performed which resulted in persistently positive margins. After two weeks of treatment (POD46), fungal susceptibilities resulted exhibiting azole resistance with amphotericin B minimal inhibitory concentration (MIC) of 4 mcg/mL (Table 4). Due to these results, amphotericin B was increased to 5 mg/kg and oral terbinafine 500 mg every 12 hours was added. Additionally, liposomal amphotericin B 250 mg/500 mL OR irrigation dwells for 30 minutes were performed by a four-quadrant wash with abdomen oscillation with each dwell taking place 5 days a week. After a total of 8 OR amphotericin B irrigation dwell completions and continued systemic combination antifungal therapy, the patient's abdominal wall was closed on POD49. On POD53, isavuconazole was switched to IV posaconazole 300 mg every 12 hours with dose adjustments per therapeutic drug monitoring (Table 3) and nystatin 100,000 unit/mL suspension to be swallowed was added. The closure of the abdomen was complicated by dehiscence and necrotic appearing tissue at the incision site. After debridement, advancement of fasciocutaneous flap, and incisional wound vacuum placement, the amphotericin dose was transitioned to 7.5 mg/kg three times a week in preparation for discharge. Amphotericin B was continued outpatient until POD213 and posaconazole for a year from discharge. The patient remained *Rhizopus* spp. free at 1-year postdiagnosis.

### 2.3. Patient 3

The last patient is a 62-year-old Caucasian female with a past medical history of hypertension and coronary artery disease who underwent an OLT secondary to chronic HCV infection and alcoholic cirrhosis. MELD-Na at time of transplant was 51. Overall, the patient tolerated the procedure well. Due to intra-abdominal hemorrhage from ongoing coagulopathy, the patient's abdomen was packed with a planned second operation for completion of the biliary anastomosis on POD2. Immunosuppression with a corticosteroid taper, tacrolimus, and mycophenolate mofetil was started on the day of transplant per institution protocol (Table 1). Infection prophylaxis consisted of sulfamethoxazole/trimethoprim (POD3), valganciclovir (POD1), and voriconazole (started 24 days prior to transplant). Hepatitis B total core antibody resulted positive, and tenofovir disoproxil was initiated POD6.

**Figure 3:**
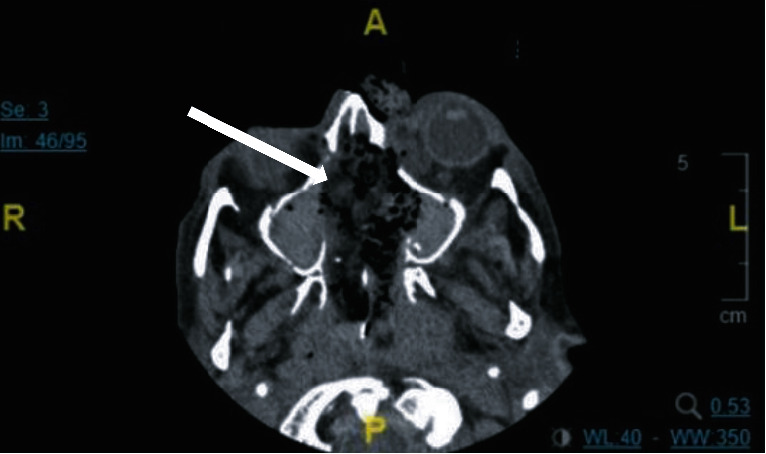
Patient 3 MRI image mucromycosis growth in sinuses.

**Figure 4:**
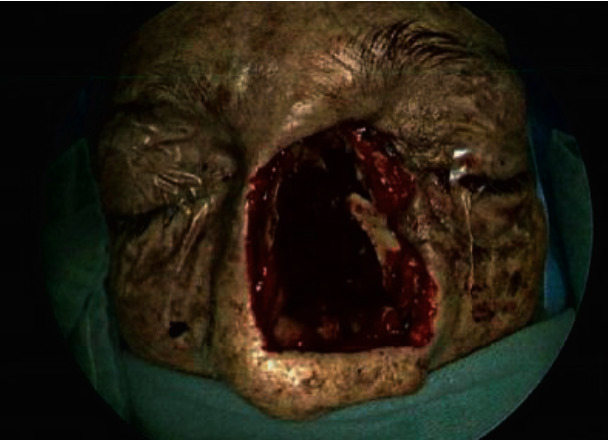
Patient 3 postsurgical intervention.

On POD6, empiric antimicrobials were broadened in response to increased leukocytosis. A minibronchoalveolar lavage was performed on POD8, and cultures resulted mould on POD 10. Prophylactic voriconazole was increased to treatment dosing, and inhaled amphotericin B was started. POD12 observation of a lesion on the patient's nose raised suspicion for mucormycosis, and that same day, the cultures resulted in *Rhizopus* spp. Triple antifungal therapy was initiated (IV liposomal amphotericin B 7.5 mg/kg, posaconazole 300 mg every 12 hours, and micafungin 150 mg daily). MRI findings showed persistent marked pansinusitis with focal left nasal bridge ulceration and extension of inflammation into the postseptal inferomedial extraconal fat of the left orbit with no acute intracranial abnormality identified (Figure 3). In POD13, the patient underwent extensive surgical intervention including a partial rhinectomy, bilateral endoscopic sinus surgery with left medial maxillectomy, resection of intranasal contents, septectomy, inferior and middle turbinate resection, bilateral ethmoidectomy, right maxillary antrostomy, and resection of left lamina papyracea (Figure 4). Multiple returns for debridement occurred until the patient was found to have extensive necrotic tissue involving the skull base. Given involvement of the skull base, further debridement was stopped as this was unresectable. Susceptibilities resulted showing azole resistance and amphotericin B MIC of 0.25 mcg/mL (Table 5). Due to limitations of further surgical debridement and continued positive biopsy samples for 5 months postdiagnosis despite triple antifungal therapy, nasal packing was impregnated with amphotericin B deoxycholate 50 mg (1 mg/mL) and exchanged every 24 hours to accomplish a higher, localized concentration to combat the lack of tissue perfusion. The amphotericin B nasal packing was in addition to the triple antifungal therapy previously described. Three weeks after initiation of amphotericin B nasal packing, an obtained biopsy sample resulted negative for invasive fungal disease. Nasal packing was discontinued 4 days after negative results, and triple antifungal therapy was continued. The patient was discharged to a long-term acute care hospital with continuation of dual antifungal therapy (amphotericin 7.5 mg/kg daily and oral posaconazole 300 mg every 12 hours) with plan for follow-up reconstruction surgery. The patient remained *Rhizopus* spp. free at 1-year postdiagnosis.

## 3. Discussion

We present three cases of *Rhizopus* spp. invasive fungal infection in OLT recipients with high MELD-Na at time of transplant. In treatment of these infections, surgical intervention is imperative. Mortality has been shown to double in patients who received systemic antifungal therapy alone versus in addition to surgical intervention (55% vs. 27%) [2]. Aggressive surgical intervention to remove necrotic tissue is crucial to limit the spread of this rapidly evolving infection. Not only does this limit the spread but also helps to remove the necrotic tissue which is problematic for systemic antifungal success. The necrotic tissue exhibits decreased blood flow to the site of infection thus in turn results in a decrease in systemic antifungal concentrations. To further increase infection site antifungal concentration, unique strategies were employed in our three patients. All three patients received amphotericin B locally in the form of wound vacuum irrigation, abdominal irrigation dwells, or nasal packing, respectively (Table 6). Amphotericin B is nephrotoxic requiring pre- and posthydration. This strategy provides high concentrations at the target site as well as possibly limits nephrotoxicity. The localized therapy allowed the treatment team to achieve high concentrations of amphotericin B without an increase in the IV dosage. We believe this was a major component of the therapy plan that led to successful treatment in these 3 patients.

Another strategy utilized was the combination of 3 systemic antifungal agents. All 3 patients were treated with amphotericin B, an azole, and an echinocandin. No prospective randomized studies for optimized treatment of mucormycosis have been performed due to the rarity and heterogenous nature of these infections [2]. Amphotericin B is the cornerstone of systemic antifungal treatment of mucormycosis as this agent has reliable activity. Posaconazole and isavuconazole are both active against *Rhizopus* spp.; however, interestingly all 3 of our patients had strains that were resistant to isavuconazole (MIC ≥16 *μ*g/mL). The addition of echinocandins is controversial in literature. A case-control study completed at MD Anderson Cancer Center investigated early liposomal amphotericin B monotherapy versus combination therapy. The most common combination therapy received was liposomal amphotericin B plus an echinocandin at 46%, with posaconazole or triple therapy following behind at 27% each. However, the authors did not find any survival benefit for combination therapy over early administration of amphotericin B [4]. Echinocandins have an overall benign side effect profile and *Rhizopus* spp. have been shown to express the site of action, *β*(1,3)-D-glucan, making triple therapy a reasonable possibility in treatment of these aggressive infections [5].

The last treatment strategy performed in our patients was reduction of immunosuppression (Table 1). Our patients were high MELD-Na recipients receiving immunosuppression at time of diagnosis of the mucormycosis. To aid the antifungal therapy plan, net state of immunosuppression was decreased to enable the host's immune response to strengthen. This strategy must weigh the risk versus benefit with the possibility of transplant organ rejection. Of note, azole antifungals inhibit CYP3A4 in the liver which is a major component of drug metabolism in the human body. This can lead to the possible side effect of hepatotoxicity and drug-drug interactions. It is important to consider this when starting azole antifungals and adjusting immunosuppression as tacrolimus is metabolized by CYP3A4. These drug-drug interactions present an additional challenge in the treatment of mucormycosis, but with vigilant monitoring can be administered safely.

In conclusion, mucormycosis is an aggressive fungal infection with a high mortality rate without equally aggressive treatment. Although surgical intervention is a mainstay of therapy, other additional strategies such as localized antifungal concentrations, triple antifungal therapy, and a decrease in immunosuppression can be beneficial with successful outcomes in orthotopic liver transplant recipients with mucormycosis.

## Figures and Tables

**Table 1:** Immunosuppression induction and maintenance therapy.

	Patient 1		Patient 2		Patient 3	
	Induction^1^	Maintenance	Induction	Maintenance	Induction	Maintenance
Immunosuppression regimen prior to mucormycosis treatment	Steroids^2^	(1) Tacrolimus (2) Mycophenolate mofetil 1000 mg every 12 hours (3) Prednisone 10 mg daily	Steroids^2^	(1) Tacrolimus (2) Mycophenolate mofetil 1000 mg every 12 hours (3) Prednisone 15 mg daily	Steroids^2^	(1) Tacrolimus (2) Mycophenolate mofetil 500 mg every 12 hours (3) Prednisone 20 mg daily
Immunosuppression regimen during mucormycosis treatment	(1) Tacrolimus (2) Mycophenolate mofetil 250 BID (3) Prednisone 5 mg daily		(1) Tacrolimus (2) Mycophenolate mofetil held (3) Prednisone 5 mg daily		(1) Tacrolimus (2) Mycophenolate mofetil held (3) Prednisone 5 mg daily	
Targeted FK506 level before and during mucormycosis treatment	Before: 8–10 ng/mL During: 4–6 ng/mL		Before: 6–8 ng/mL During: 3–5 ng/mL		Before: 6–8 ng/mL During: 3–5 ng/mL	

Oral medication formulation unless otherwise noted. ^1^Simultaneous liver-kidney transplant 20 months prior to mucormycosis event. ^2^Steroid induction followed by taper per institutional protocol: IV methylprednisolone 500 mg POD0, 200 mg POD1, 160 mg POD2, 120 mg POD3, 80 mg POD4, 40 mg POD5, and then prednisone 20 mg PO daily.

**Table 2:** Patient 1 susceptibilities.

Susceptibility report Fungus culture isolate: *Rhizopus* spp.	Specimen site: leg Specimen source: tissue
Amphotericin B	0.25 *μ*g/mL
Itraconazole	≥16 *μ*g/mL
Posaconazole	4 *μ*g/mL
Voriconazole	≥16 *μ*g/mL
Isavuconazole	≥16 *μ*g/mL

**Table 3:** Therapeutic drug monitoring results of posaconazole.

Posaconazole, quantitative by LC-MS/MS		
Patient 1	HD27 HD29 HD41 HD53 HD62 HD64 HD71 HD75	0.4 *μ*g/mL 0.7 *μ*g/mL 1.1 *μ*g/mL 0.6 *μ*g/mL 0.7 *μ*g/mL 0.4 *μ*g/mL 1.2 *μ*g/mL 1.3 *μ*g/mL
Patient 2	POD38 POD62 POD76	0.6 *μ*g/mL 0.6 *μ*g/mL 1.7 *μ*g/mL
Patient 3	POD21 POD27 POD53 POD54 POD126	0.8 *μ*g/mL 0.7 *μ*g/mL 2.7 *μ*g/mL 1.7 *μ*g/mL 0.9 *μ*g/mL

HD: hospital day; POD: postoperation day.

**Table 4:** Patient 2 susceptibilities.

Susceptibility report Fungus culture isolate: *Rhizopus* spp.	Specimen site: stomach Specimen source: tissue
Amphotericin B	4 *μ*g/mL
Itraconazole	≥16 *μ*g/mL
Posaconazole	≥16 *μ*g/mL
Voriconazole	≥16 *μ*g/mL
Micafungin	≥8 *μ*g/mL
Isavuconazole	≥16 *μ*g/mL

**Table 5:** Patient 3 susceptibilities.

Susceptibility report Fungus culture isolate: *Rhizopus* spp.	Specimen site: nose Specimen source: lesion
Amphotericin B	0.25 *μ*g/mL
Itraconazole	≥16 *μ*g/mL
Posaconazole	≥16 *μ*g/mL
Voriconazole	≥16 *μ*g/mL
Isavuconazole	≥16 *μ*g/mL

**Table 6:** Summary of presented cases.

Patient	Site of infection	Systemic therapy	Local therapy	Surgical therapy
1	Left lower extremity	(1) Isavuconazole 372 mg daily then switched to posaconazole 300 mg daily (2) Liposomal amphotericin B 3 mg/kg (3) Micafungin 150 mg daily	Amphotericin B deoxycholate 50 mg/1000 mL irrigation through wound vacuum	Fasciotomy and debridement
2	Abdominal	(1) Isavuconazole 372 mg every 8 hours for 2 days then 372 mg daily (2) Liposomal amphotericin B 3 mg/kg daily (3) Micafungin 150 mg daily	Liposomal amphotericin B abdominal irrigation dwells	Serial OR debridement and washouts
3	Sinuses	(1) Posaconazole 300 mg every 12 hours (2) Liposomal amphoteric B 7.5 mg/kg (3) Micafungin 150 mg daily	Amphotericin B deoxycholate 1 mg/mL nasal packing	Rhinectomy, resection of intranasal contents, and multiple debridement sessions

Initiation date of therapy included in case series body. OR: operational room.

## Data Availability

Data is not publicly available but was collected retrospectively under approval from the Institutional Review Board.
